# Hypertransaminasemia and hypophosphoremia in an adolescent with anorexia nervosa: an event to watch for

**DOI:** 10.1186/s13052-016-0258-3

**Published:** 2016-05-17

**Authors:** Maria Rosaria Marchili, Elena Boccuzzi, Anna Chiara Vittucci, Lelia Rotondi Aufiero, Stefano Vicari, Alberto Villani

**Affiliations:** Bambino Gesù Children’s Hospital, Rome, Italy

**Keywords:** Anorexia nervosa, Refeeding syndrome, Hypercholesterolemia in AN, Hypertransaminasemia in AN

## Abstract

**Background:**

Anorexia Nervosa is a Psychiatric eating disorder of adolescence age with a high morbidity and mortality.

**Case Presentation:**

We describe a common case of anorexia nervosa (AN) in a female adolescent complicated by less known conditions related to refeeding syndrome. At admission, the girl showed a mild hypercholesterolemia with progressive normalization of the values. The initial low hypertransaminasemia worsened after refeeding until very high levels and hypophosphoremia was also described. Only a controlled caloric intake and a specific electrolyte supplementation led to the improvement of hematologic values and the clinical condition of the patient.

**Conclusions:**

Refeeding complications must be always suspected because of life-threatening risk. More attention should be paid not only to the acute state of the disease but also to the prevention and the management of refeeding-related manifestations.

## Background

Anorexia Nervosa (AN) is a psychiatric eating disorder with a high morbidity and mortality rate that mainly affects adolescent females aged 15 to 19 years [1]. In malnourished patients depletion of fat and fat free mass lead to reduced energy expenditure, cardiovascular alteration and metabolic adaptation to starvation (ability to function in a hypometabolic state) so even at advanced stages biochemical presentation of the disease is often unremarkable. Among the few abnormal laboratory values, hypercholesterolemia can be found despite emaciation and avoidance of cholesterol-rich food. Several hypotheses have been proposed but the improvement of serum levels after refeeding mainly confirms the hypothesis of accelerated cholesterol metabolism in the fasting phase of AN [2]. The prevalence of a mild increase in serum transaminases levels in AN patients prior to refeeding range widely and it is observed in up to 76 % [1, 3]. Marked increases (>200 UI/L) are less common; an inverse relationship between aminotransferase levels and body mass index (BMI) suggests that nutritional states play a role in liver changes of these patients [4, 5]. In fact it occurs when BMI reaches a critically low level [6], particularly less than 12 kg/m^2^ [7]. Several factors can contribute to determining hypertransaminasemia but the main understanding mechanism seems to be autophagy that is a physiological hepatoprotective event activated by cells during episodes of stress, such as fasting [6]. It leads to elevation of aminotransferases levels because of increased permeability of the cytoplasmic membrane allowing the release of transaminases in to the blood without histological necrosis [6].

The treatment of AN is complex involving a multidisciplinary approach. Regaining weight is one of the major factors predicting favorable short and long term outcomes, also associated with improvement in psychological and medical complications [1]. However refeeding of malnourished patients is not free of complications: the refeeding syndrome (RS) is the most frequent one [8] characterized by severe intracellular electrolyte shift with consequent low-serum electrolyte concentrations, acute circulatory fluid overload and organ failure [9]. The cause of RS is an excess or unbalanced oral, enteral or parenteral nutritional intake [9] and the risk of its development is highest during the first week [10]. According to NICE guidelines (2004) the patients in greatest danger have a BMI <16 kg/m^2^.

The hallmark of the RS is a severe hypophosphoremia [9] that is more likely to occur in severely malnourished patients [10]. Its prevalence is from 28 up to 45 % during refeeding, but literature shows that lower incidence (about 5 %) is reported in studies conducted on patients who were given electrolytic supplementation despite normal levels at admission [3] showing how its empirical administration could reduce the incidence of electrolyte anomalies. An increase of transaminases levels after initiating refeeding frequently occurs [7]. Such an increase is commonly referred to as steatosis that can be considered the hallmark of refeeding related hepatic dysfunction and can occur with any form of feeding. The suspected mechanisms are excess oral glucose administration, excessive exposure of the liver to pro-inflammatory cytokines and excess free fatty acid exposure [7]. A liver ultrasound may be helpful to distinguish starvation-induced hypertransaminasemia (normal ultrasound) where continued feeding is indicated from refeeding-induced hypertransaminasemia (steatosis on ultrasound) where a caloric reduction and a change of feeding are needed.

## Case presentation

**Fig. 1:**
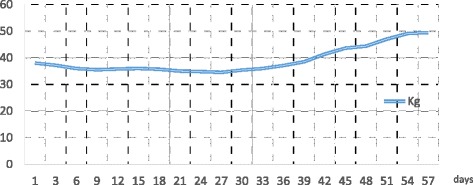
Weight trend during hospitalization. The weight is expressed in kilograms (kg). The hospitalization is expressed in days of recovery on the X axis

**Fig. 2:**
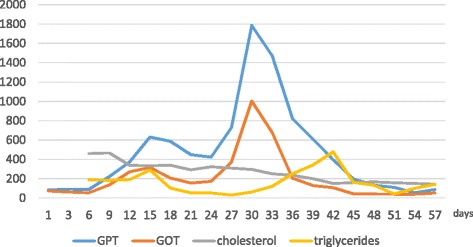
Transaminases, cholesterol and triglycerides trends during hospitalization. The transaminases (GPT: Glutamic Pyruvic Transaminase; GOT: Glutamic Oxaloacetic Transaminase) are expressed in UI/L, cholesterol and triglycerides in mg/dl. The hospitalization is expressed in days of recovery on the X axis

**Fig. 3:**
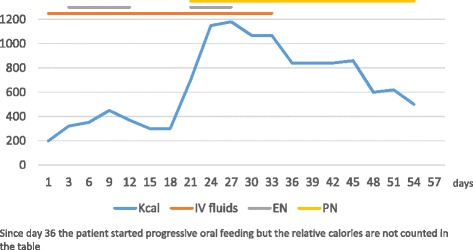
Caloric intake variation during hospitalization related to refeeding way. The hospitalization is expressed in days of recovery on the X axis. The refeeding ways reported on the top of the figure are Enteral Nutrition (EN) and Parenteral Nutrition (PN), even if also intravenous (IV) fluids have been administered during more than one half of the hospitalization. The calculated caloric intake does not include the share of the oral one started at day 36 of hospitalization

We report of a 15 year-old girl admitted to the Department of Pediatric Medicine for bradycardia in anorexia nervosa. The onset of her medical history dates back 12 months before admission when the girl started dieting under medical supervision because she was overweight (90 Kg. BMI 33.5 kg/m^2^). Despite the attainment of target weight she continued the diet with a progressively more marked restriction of food intake until she weighed just 38 Kg with BMI of 14 kg/m^2^, representing 63 % of her ideal body weight. Upon admission, the patient was cachectic and she had severe bradycardia (heart rate of 35 bpm), blood pressure of 80/60 mmHg, hypothermia, dry skin, subcutaneous emphysema on the neck and shoulders, constipation and amenorrhea for 5 months. The girl showed marked mood deflection with a high level of generalized anxiety and distorted perception of body image with an intense fear of gaining weight. The blood examinations showed: GOT 61 UI/L (normal range, 5–40 UI/L), GPT 92 UI/L (normal range, 5–40 UI/L), total cholesterol 460 mg/dl (normal range, 140–200 mg/dl) and triglycerides 188 mg/dl (normal range, 60–170 mg/dl). Abdominal ultrasound was normal, as well as chest X-ray. After a careful multidisciplinary assessment the girl underwent intravenous rehydration (with 5 % glucose solution, sodium and potassium supplementation) and both psychological support program and antipsychotic therapy with aripiprazole and fluoxetine were started. Despite this approach the patient kep on refusing food and losing weight so she was subjected to forced enteral feeding with nasogastric tube. Over the following weeks blood tests showed gradual worsening of hypertransaminasemia. Infections, autoimmune conditions, metabolic diseases and iatrogenic causes were investigated and excluded. In particular, even if prescribed antipsychotic therapy could be potentially involved in increasing of transaminase levels, its role was excluded because of the low dose (aripiprazole at 2.5 mg/day and fluoxetine at 10 mg/day) and the lack of improving after its discontinuation. The weight reached a minimum of 35 kg and did not improve (Fig. 1). Enteral nutrition (EN) was reduced and parenteral nutrition (PN) was later started with a significant increase of both caloric intake (until 35 kcal/kg/d) and transaminases. The liver function tests showed maximum values of GOT 1003 UI/L and GPT 1784 UI/L after about a month of hospitalization (Fig. 2) and signs of hepatic steatosis on the ultrasound were simultaneously described. The subcutaneous emphysema was gradually reduced but the onset of bilateral pretibial edema was observed. Ten days after the beginning of PN severe hypophosphoremia (0.4 mg/dl; normal range, 2.7–4.5 mg/dl) and isolated macrocytic anemia (Hb 6.9 mg/dl and MCV 100 fl) were observed. This evidence, associated with the persistence of hypertransaminasemia and peripheral edema, led to suspicion of refeeding disorder. So a progressive restriction of caloric intake (Fig. 3) and a specific electrolyte and vitamin supplementation were implemented with subsequent gradual improvement of blood values and clinical condition of the patient. After about two months of hospitalization a satisfactory weight of 45 Kg (BMI 16.8 kg/m^2^) was reached. At now, one year after discharge from hospital, the girl again weighs 85.7 Kg (BMI 30.8 kg/m^2^). Despite the periodic clinical and neuropsychiatric evaluations, the eating disorder has not been overcome.

## Conclusion

None of the laboratory or clinical aspects of our case remain unexplained neither those ralated to disease nor those related to the complications of refeeding. In particular, the hypercholesterolemia and the hypertransaminasemia we observed at admission are both related to starvation and well described in literature. After refeeding the former improved progressively and the latter worsened because of the establishment of mechanisms related to the refeeding and described previously. However, as observed in several studies, it gradually normalized after restriction of caloric intake. The only aspect of hyperaminotransferasemia that differs from scientific evidence is related to the time of its onset. The patient had a delayed increase of liver function tests with maximum values a month after admission and forced refeeding, contrary to the earlier onset reported in literature. We are not able to give a complete explanation of this data; probably it is due to the caloric intake being increased to quickly as a result concomitant EN and PN. Furthermore this nutritional approach didn’t allow us to distinguish the role of enteral caloric intake from the parenteral one. Regarding macrocytic anemia, the normalization of the values after supplementation of folate and B12 vitamin which was followed by the gradual regularization of diet later on, supports the hypothesis of their deficiency even if their hematic levels were not investigated. Finally hypophosphatemia is not a rare condition because it is the hallmark of the RS and it must always be investigated in all patients who undergo refeeding in order to perform a specific electrolyte supplementation and a possible change of diet plan, as we did. However the step prior to the management of hypoP could be its prevention even if no guidelines exist for prophylactic phosphate dosing [11].

Weight gain is the final target of refeeding and it is also associated with shorter hospitalization. However many authors confirm a direct link between energy intake, hypophosphoremia and other refeeding disturbances, underscoring the need to identify approaches that simultaneously maximize weight recovery and minimize associated risks. The best way to balance such aspects is the early identification of high risk patients. In particular ideal body weight percentage at admission (<70 % of expected body weight) is predictive of the development of hypoP [11, 12].

For many years an initial low calorie approach and a slow increase have been recommended [1, 8] even if different ranges of energy intake have been proposed [1, 2] particularly at the beginning, and there is lack of consensus about the best way to administer nutritional intake. Recently, different studies report a higher caloric intake, particular in mildly and moderately malnourished patients. However a lower caloric approach is still recommended for severely malnourished patients (BMI < 15 kg/m^2^) because of the higher risk of complication of refeeding [10]. Until now, the lack of consensus and ambivalence about appropriate refeeding intake remain inconsistent and guidelines seem to be based on clinical experience rather than scientific evidence [8]. So it is not possible to suggest any initial energy intake prescription as the safest, most efficient or preferable but the evaluation of nutritional status of patients at admission, the identification of risk factors for RS and the rapid identification of complications must be considered to allow us to manage the patient as best we can. Individualized diet plan based on standardized indications is advisable for patients with AN even if it is described that patients receiving standardized treatment have an accelerated weight gain without increasing of the incidence of RS compared to persons receiving individualized caloric prescriptions [13]. So, further studies are mandatory to define such issue. Finally, attention should be paid to the follow-up of these fragile patients even after discharge to prevent the recurrence of eating disorders which is not unlikely, as in our case.

## Consent for publication

Consent to publish was obtained from patient’s parent.

## Abbreviations

AN: Anorexia nervosa
EN: Enteral nutrition
PN: Parenteral nutrition
RS: Refeeding syndrome
